# Deep Conviction Systems for Biomedical Applications Using Intuiting Procedures With Cross Point Approach

**DOI:** 10.3389/fpubh.2022.909628

**Published:** 2022-05-23

**Authors:** Hariprasath Manoharan, Shitharth Selvarajan, Ayman Yafoz, Hassan A. Alterazi, Mueen Uddin, Chin-Ling Chen, Chih-Ming Wu

**Affiliations:** ^1^Department of Electronics and Communication Engineering, Panimalar Institute of Technology, Chennai, India; ^2^Department of Computer Science & Engineering, Kebri Dehar University, Kebri Dehar, Ethiopia; ^3^Department of Information Systems, Faculty of Computing and Information Technology, King Abdulaziz University, Jeddah, Saudi Arabia; ^4^School of Digital Science, University Brunei Darussalam, Bandar Seri Begawan, Brunei; ^5^School of Information Engineering, Changchun Sci-Tech University, Changchun, China; ^6^Department of Computer Science and Information Engineering, Chaoyang University of Technology, Taichung, Taiwan; ^7^School of Computer and Information Engineering, Xiamen University of Technology, Xiamen, China; ^8^School of Civil Engineering and Architecture, Xiamen University of Technology, Xiamen, China

**Keywords:** biomedical signals, deep learning, Fourier filters, sensors, cross points

## Abstract

The production, testing, and processing of signals without any interpretation is a crucial task with time scale periods in today's biological applications. As a result, the proposed work attempts to use a deep learning model to handle difficulties that arise during the processing stage of biomedical information. Deep Conviction Systems (DCS) are employed at the integration step for this procedure, which uses classification processes with a large number of characteristics. In addition, a novel system model for analyzing the behavior of biomedical signals has been developed, complete with an output tracking mechanism that delivers transceiver results in a low-power implementation approach. Because low-power transceivers are integrated, the cost of implementation for designated output units will be decreased. To prove the effectiveness of DCS feasibility, convergence and robustness characteristics are observed by incorporating an interface system that is processed with a deep learning toolbox. They compared test results using DCS to prove that all experimental scenarios prove to be much more effective for about 79 percent for variations with time periods.

## Survey of Conventional Models

As many researchers have contributed their works for changing advancement that is associated with real-time effects, this part describes and contrasts numerous models that are related to developments in the biomedical field industry. Several modes of operation have been investigated even in past generations without the use of computer-aided design, and the end-stage results are insufficient for confirming the effective models. As a result, the colloquium began with a communication and signal processing toolbox (1), which uses a machine learning technique with a complicated dynamic nature and distributed optimization. However, in all case studies, a random technique is used, with a focus on sixth-generation networks, and even high sensing conditions have been made practical using these types of ideal procedures. A comprehensive review strategy is established using several case studies, including neuron science and cardiovascular image processing techniques, where significant influence has been demonstrated under various algorithmic settings (2). However, because analytic information is required for all representation scenarios, photos are not processed using the training model. Even though 60% of the reference model is operational, the presentation of biological disease with various picture models cannot be compared under extreme situations.

Aside from the case studies stated above, a deep learning method with high feature extraction has been shown to be an effective technique for geometric scale analysis (3). The main drawback of geometric scale analysis is that dynamic state measurements do not work with baseline health data because differences are noticed at stage 2. Even feature state analysis has been investigated using weight transformations, and such state changes have been shown to operate with a variety of deep representations. However, data augmentation using end networks only works when there are numerous learning problems (4), which are not present in this type of deep learning technique. Furthermore, the physiological expression of signals is categorized using random value creation, and the values are observed, resulting in heavy noisy data (5). As signals are reconstructed as the value pushes lower down to the source point, this noisy data will have a significant impact on biomedical signals, with more physiological sources having computational consequences as signals are recreated. As morphological data is varied throughout separate time intervals, the aforementioned drop in source point can be convalesced using a soft max function. Data will be split into five separate forms for soft max functions, enhancing the capability of the abstraction process.

Furthermore, the firefly algorithm (6) can be used to handle issues and challenges linked to biological applications, where all health problems are studied in-depth, resulting in creative answers through data fusion. Many parametric measures are still displaced due to insufficient access information from other networks, necessitating realistic experimentation with data generated from a centralized perspective. After detecting minimal parametric changes in the firefly method, real-time signal generator algorithm updates are made with incoming signal validation (7). Protocols are also framed for the generation of algorithms using the layered decomposition technique, which allows signals to be partially communicated. This partial signal transfer will be accomplished using a decoding technique that is based on binary values. As a result, a unique formulation is required for converting a standard arrangement to a binary system, which can be accomplished using training procedures seen in transmission lines with epoch iterations. Despite the fact that real-time algorithms are more efficient than 70% of the time, data sets are not established in a good initialization model, thus a primate algorithm with high extraction features and parametric stabilizations is used with a longer overall testing time (8). Due to the fact that stabilizations are prepared in a dual mode of operation, relative errors are further lowered to optimum values, which on average fall below 82. Despite the intricacy of temporal representations, two different data sets will be presented, enhancing the risk of misinterpretation during extensive analysis.

Furthermore, biological signals are integrated at a massive scale utilizing gate count technology (9), and various signals are handled using system architectures. The operating frequencies of various signals are not determined due to the presence of diverse designs, resulting in a miscount of separate integration gates. As a starting point, neural networks are useful for processing crucial signals because they take advantage of a variety of compositional properties of biological points of representation (10). Furthermore, if any problems are found in a large structure, ECG and EEG data will be isolated, allowing for future orientations and change. Between 2012 and 2018, there were numerous techniques that used sensor equipment and permitted the feel and touch method during the communication process (11). Electroencephalography data are evaluated in this feeling, which is modeled as a new technique with seven distinct layers. In accordance with the aforementioned issue, the 7-layer technique is carried out in parallel units, with programming written at high rates employing processor units. Many advantages can be seen for biomedical applications with such high rates, where justification can be processed before pre-processing phases (12–14). These advantageous systems are created using wearable devices where auto-learning models are activated. This type of automated process will allocate signals that move at different frequency periods with a classification mechanism (15). However, it is much easier to separate different signals at unique frequency periods but a wearable device must be created for separating the individual monitored values rather than signals. Further biomedical signals are tested for the prediction of cardiovascular disease using conventional neural networks where a short-term memory coder is implemented in the system (16, 17). The major disadvantage is that if signals are processed, then high memory is required for storing the information that can be accessed at later stages.

### Research Gap and Motivation

All the different approaches (1–14) that are discussed using different methods for processing biomedical signals which are not accurate in the field of medical diagnosis. The biomedical signals cannot be passed without the presence of proper communication units at both the transmitter and receiver sides. However few efforts (15–17) have been made by some researchers to prove the complete effectiveness of biomedical signal processing in various applications and they have achieved 63% of accuracy in considered cases. Therefore, the proposed method witnesses the abovementioned gap and provides a solution for effectively utilizing biomedical signals in medical applications. The process functions using a Deep Conviction Systems (DCS) which is an automated procedure for capturing the image of a particular individual using low power technology. This type of implementation system reduces the risk of implementation as high resistance is provided with tracking mechanisms. Also, the data gathering path using biomedical signals is faster and it is replaced with some units in the existing system. This adds an advantage as the entire system is built with low-cost networks that provide more than 80% accuracy on the input side even if the signal strength is at the intermediate stage.

### Objectives

The proposed work on biomedical signal processing is a three-stage objective process that aims to solve the following,

- To incorporate a pre-processing stage that incarcerations the input images to a small matrix type
- To maximize the distance of measurement by transmitting the biomedical signals with recorded output type
- To generate a data augmentation procedure for apprehending the information that is processed using DCS

## System Model: Geometric Analysis of Signals

In this section, the system model that supports the generation of signals with respect to algebraic equations is framed using two types of instability models. The major reason for selecting unsteady models is that the signals will be generated using the spectral mode of transmission and it varies for several operating cases. Thus, a segment of sensors is integrated for monitoring huge variations of input signals therefore all recorded output signals will be represented using a non-specific Equation which is represented below,


(1) $$\begin{array}{l} d_{a}\left(t\right)=\sum_{i=1}^{n}s_{t}\;(i)+\;n_{i}\left(t\right) \end{array}$$

Where,

*d*_*a*_(*t*) indicates the acquired data that changes with time periods

*s*_*t*_ (*i*) and *n*_*i*_ (*t*) represents true stable and noisy signals respectively

From Equation (1) the noisy signals will have Fourier filters as discrete noise from individual signals is divided with time period representations. Further filters are applied in the case where different time scales are observed for periodic scales as biomedical signals are supplied with a data attainment approach. Thus the time scales can be varied using Equation (2) as follows,


(2) $$\begin{array}{l} t_{i}=\sum_{i=1}^{n}d_{a}\left(t-\frac{s_{t}}{2}\right)\left(t+\frac{s_{t}}{2}\right) \end{array}$$

Where,

$t-\frac{s_{t}}{2}$ and $t+\frac{s_{t}}{2}$ represents positive and negative time samples

Equation (2) is represented in the form of significant variant properties thus the concession matrix can be used for normalizing all the connected signals. This can be represented in mathematical form as follows,


(3) $$\begin{array}{l} \rho_{i}=\frac{1}{2}\sum_{i=1}^{n}w_{i}|c_{in}| \end{array}$$

Where,

*w*_*i*_ denotes weight factor of applied bio-signals

*c*_*in*_ indicates the number of connected networks in the signaling process

The connected networks in the signal processing technique will have upper and lower boundaries which are represented using shifting boundaries using Equation (4) as follows,


(4) $$\begin{array}{l} c_{in}=\sum_{i=1}^{n}\frac{u_{in}\;(t)+l_{in}\left(t\right)}{Total\;period} \end{array}$$

Where,

*u*_*in*_ (*t*) and *l*_*in*_ (*t*) represents the upper and lower limits

Equation (4) is also characterized as mean values of signals and appropriate integer values cannot be obtained in any case. Therefore, the quantization of integers at each step period can be represented using Equation (5) as follows,


(5) $$\begin{array}{l} q_{i}\;(t)=\sum_{i=1}^{n}\left[\frac{min\left(in\right)}{max\left(in\right)}\right]{\;}^{*}\;\left(S_{i}-1\right) \end{array}$$

Where,

min(*in*) and max(*in*) denotes minimum and maximum quantization values of signals

*S*_*i*_ represents the data size of signals

In Equation (5) data size of signals is considered using an array of the matrix as 16^*^16 as the convolution of signals can be carried within the corresponding network frame. Subsequently, the defined array size can provide errors and it can be solved using the probability density function of signals as represented in Equation (6) as follows,


(6) $$\begin{array}{l} \sigma_{i}=\sum_{i=1}^{n}\log P_{t}\left(i\right)\left(1-\log P_{o}\left(i\right)\right) \end{array}$$

Where,

*P*_*t*_(*i*) and *P*_*o*_(*i*) represent true probability and original values of signal positions

The geometric analysis of biomedical signals is used for solving both minimization and maximization problems that can be represented using the objective function as given in Equation (7).


(7) $$\begin{array}{l} {Obj}_{i}=min\sum_{i=1}^{n}C_{in},t_{i}\;and\;max\sum_{i=1}^{n}{dist}_{i} \end{array}$$

Equation (7) indicates that two minimizations and one maximization problem will be solved using the proposed DCS algorithm for processing the biomedical signals at high strength to monitor all necessary parameters.

## Optimization Algorithm

The major advantage of DCS as compared with other non-fusion algorithms is that it is not highly robust to noise and the learning rate of DCS is much higher as compared with other algorithms. Since there is a necessity to learn and update different characteristics of biomedical signals as it varies for every time period a DCS will be much support to correct the raw data behavior (18). The process involved in DCS saves the learning time as multi-tasking is performed with automatic feature learning behavior as the structure of DCS is designed in an anatomical way (19). Deep learning methods are used for biomedical signal processing in this part because many significant features may be extracted within reasonable bounds. Even with the use of a deep learning algorithm, a wide range of complex data can be extracted using various learning patterns. As a result, one sort of deep learning algorithm known as a DCS is used since numerous learning parameters can be changed as time passes. In DCS, one visible and hidden vector is chosen at the outset, and the energy parameter is represented using a probability distribution as shown in Equation (8).


(8) $$\begin{array}{l} P\left(o_{i},s_{i}\right)=\sum_{i=1}^{n}\frac{e^{-o_{i},s_{i}}}{\omega_{i}} \end{array}$$

Where,

*o*_*i*_ and *s*_*i*_ represents the observable and secret bound values

ω_*i*_ denotes the energy representation matrix

To make use of the energy constraint, the probability values must remain at 1. As a result, it is used as a persistent significance in observable bounds, with coordinates that can be changed in the order of j. Equation (9) can be used to represent *j*^th^ order variation in expectation form as follows:


(9) $$\begin{array}{l} E\left(i,n\right)=\sum_{i=1}^{n}\frac{j_{i}}{z_{i}}\left(1-j_{i}\right) \end{array}$$

Where.

*j*_*i*_ indicates a sub-variance matrix with the change of order

In case the order in Equation (9) is recurrent then to identify the similarity index a mathematical model is framed using Equation (10) as follows,


(10) $$\begin{array}{l} j_{i}=\sum_{i=1}^{n}\frac{{\left(1-\partial \right)}^{2}}{\partial }log\left(e_{in}\right) \end{array}$$

Where,

∂ denotes similarity index of the matrix

*e*_*in*_ indicates an error in the processing of similar values

Using an unvarying distributed matrix, Equation (10) shows a logarithmic control where all comparable values can be ignored. In addition, the distance between biomedical signal processing can be determined using Equation (11) as follows:


(11) $$\begin{array}{l} {distance}_{i}=\sum_{i=1}^{n}d\;(a,b)+d\;\left(x,y\right) \end{array}$$

Where,

*a, b*, and *x, y* denote the four points of inter-section between biomedical signals

The abovementioned signals can be activated with a learning rate using maximized function values which can be defined using Equation (12).


(12) $$\begin{array}{l} r_{f}\;(i)=\{\begin{array}{c} 0\;if\text{ max}\left(0,1\right)\\ 1\;if\text{ min}\left(0,1\right) \end{array} \end{array}$$

Where,

*r*_*f*_ indicates the Relu function with maximization and minimization values

The Relu function in Equation (12) will be varied with binary values 0 and 1 using the tanh activation task as follows,


(13) $$\begin{array}{l} \tanh\left(i\right)=\sum_{i=1}^{n}\frac{e^{i}-e^{n}}{e^{i}+e^{-n}} \end{array}$$

Where,

*e*^*i*^ and *e*^*n*^ represents tasks that are performed before and after pre-processing stages

In the presence of a distinct learning rate adjustment parameters must be defined with updating of parametric values where iteration variables are used and represented using Equation (14) as follows,


(14) $$\begin{array}{l} R_{a}\;(i)=\sum_{i=1}^{n}\frac{R_{a}\;(\max)-R_{a}\left(\min\right)}{number\;of\;iterations} \end{array}$$

Where,

*R*_*a*_ indicates fine-tuning of parameters

**Figure 1:**
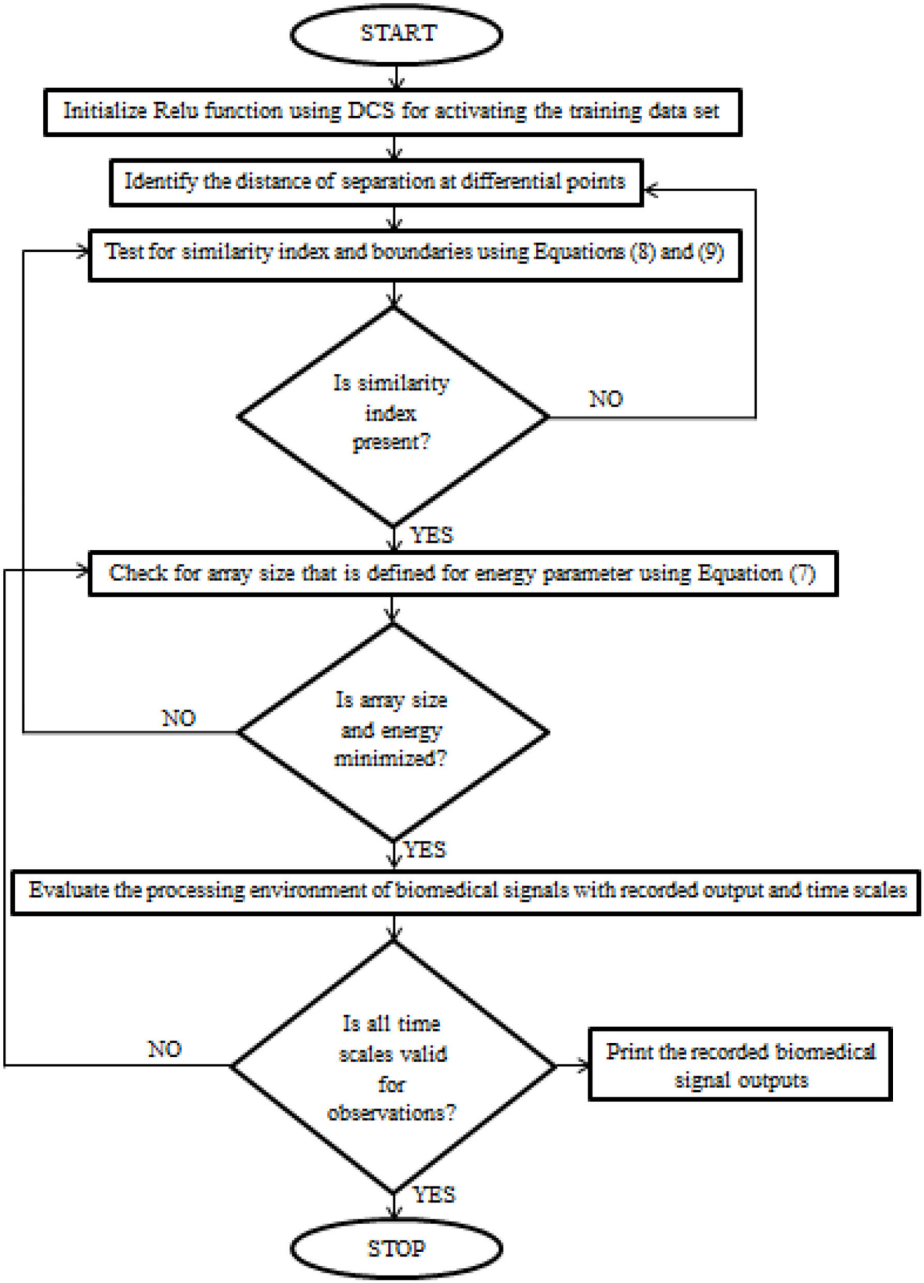
The implementation procedure of Deep Conviction Systems (DCS) for realizing biomedical signals.

After certain parametric tuning, the loop values of DCS will be produced as output with a description of the learning rate. The flow chart of DCS for biomedical signals is deliberated in Figure 1.

## Results and Discussions

In this section, the results of the system model presented in section 2 are simulated using an experimental setup consisting of Fourier filters and geometric signal processing. The experimental setting is constructed in such a way that the total number of physical quantities measured by a sensing device is minimized. Because polluted noise in biomedical signals is decreased, the sensors play an important role in the monitoring process. Both front and rear end sensors are used in the suggested method, and the compatibility of numerous signals is examined at the same time. A deep learning toolbox in MATLAB is used to convert the hardware setup to real-time simulation, with the major data being collected using a reference data set stored on the local disc. The following scenarios also show cross-validation of biomedical signal processing:

Scenario 1: Reconnaissance of recorded data
Scenario 2: Variation of time scale periods
Scenario 3: Configuration distance
Scenario 4: Task of activation
Scenario 5: Cost of implementation

All of the aforementioned situations are carried out with signals that are recorded in a hardware setup and compared to reference signals in computer-aided design. During this comparison, it is decided to use Fourier filters to separate the signals in a regular manner, hence decreasing noise. A full description of all scenarios will give you a good understanding of the seven-layer process.

### Scenario 1

**Figure 2:**
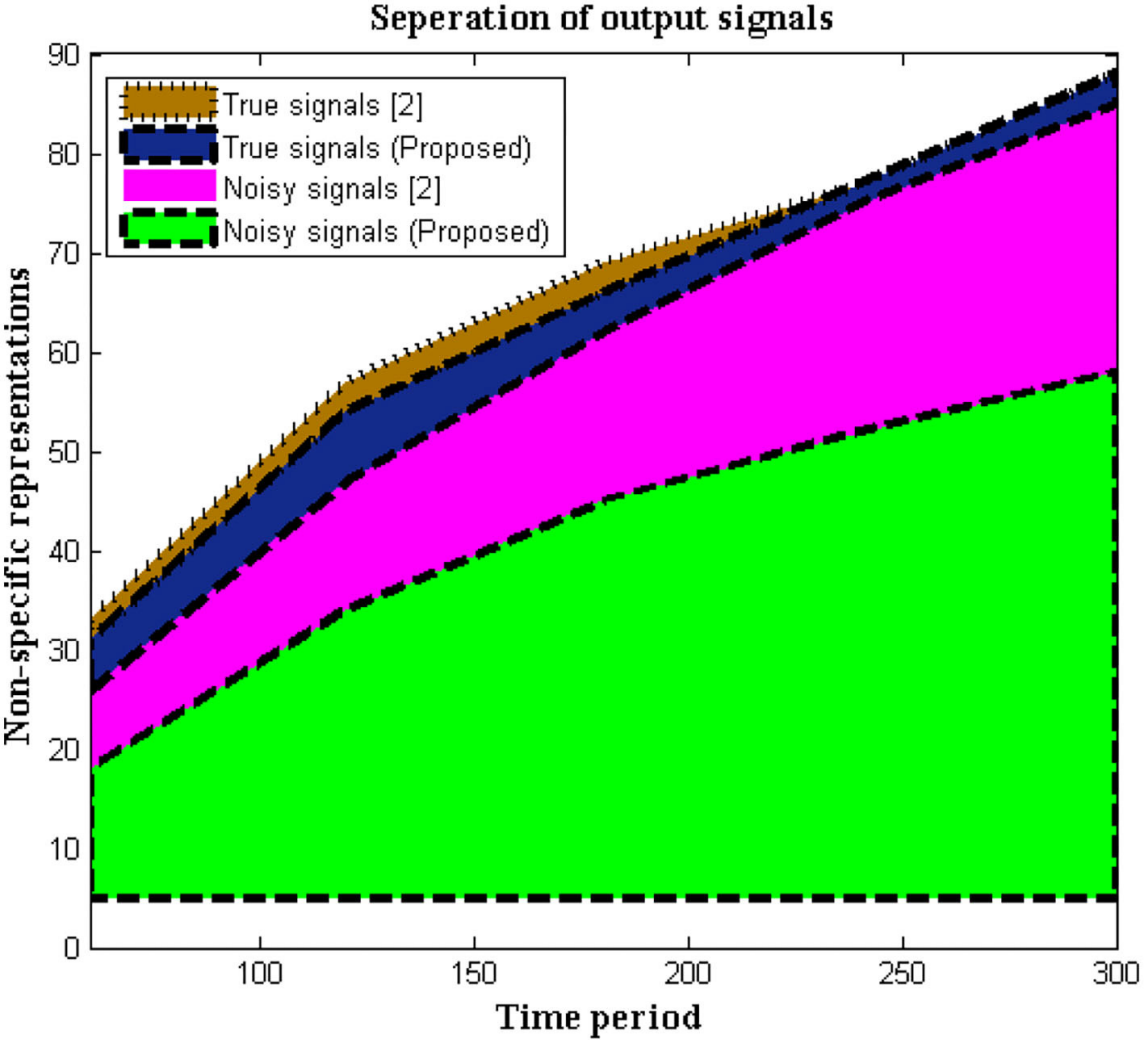
Representation of recorded data.

In this situation, the recorded data at output units is measured using non-specific time-series samples. Different dynamic ranges are identified without any labels of numerous signals, hence the output units are measured using a 16-bit sample period. Signals are delivered to the receiver without interruption because no tag action is done during the signaling interval. However, in biomedical signal processing, it is necessary to utilize tags to distinguish between real and noisy signals, as shown in Equation (1), and this may be viewed as a unique mechanism used in the execution of the suggested method. This type of separation protects the signal until it reaches the endpoint and allows users to choose channels based on their priority. If any caveat signals are recognized, time periods will be represented at a higher stage, resulting in a faster signal propagation speed. As limited appropriation is used, the above-mentioned technique is verified every 5 s. Figure 2 depicts the simulation results of recorded output unit data.

Figure 2, Table 1 show that the initial stage of representations can be achieved using these periodic representations over time durations ranging from 60 to 300 s. The number of true and noisy signals is limited throughout these periods, and the effective signal epoch boundary limits should be between 40 and 60. In addition, a comparison was done with conventional approaches using different system models, and it was discovered that the average number of real signals is formed (2), but true signal representation is determined to be at high levels in the deep learning process. In the projected technique, rather than random changes, the amount of noisy signals is reduced at the conclusion of time periods (2).

**Table 1:** Separation of stable and noisy signals.

**Time period**	**Number of stable signals (2)**	**Number of stable signals (Proposed)**	**Number of noisy signals (2)**	**Number of noisy signals (Proposed)**
60	8	18	5	2
120	13	34	7	3
180	17	45	4	3
240	23	52	2	0
300	27	58	3	0

### Scenario 2

**Figure 3:**
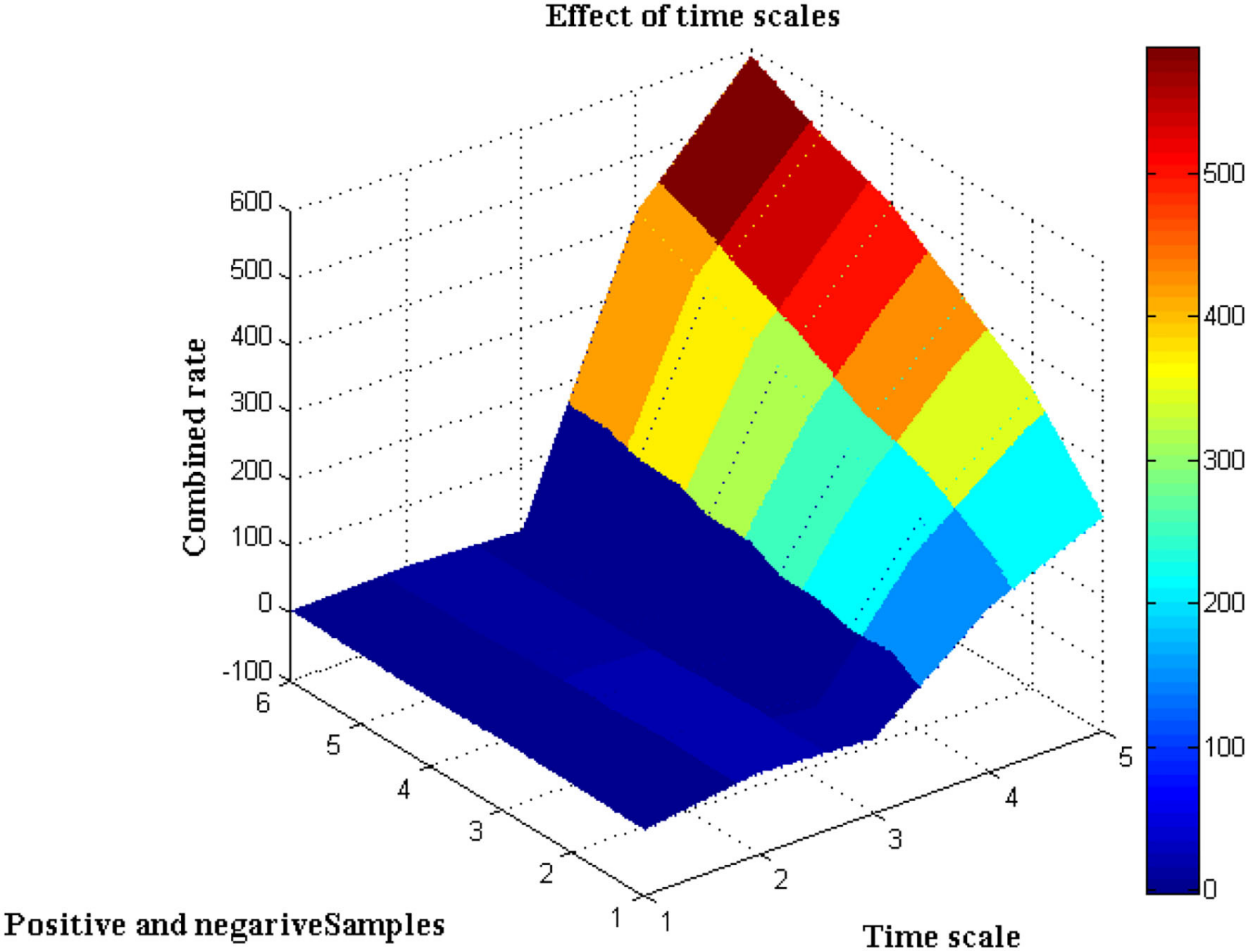
Time scale vs. Samples.

It is necessary to execute the unpredictability of time periods in addition to the separation of biological signals, which is presented in this scenario. The evolution of biological signal representation is accomplished through a stationary process in which modest changes in time periods cannot be tracked over a short period of time. However, because the suggested method produces non-stationary signals, it is possible to track and adjust the signal properties even during the automatic recording step. This benefit can be used during the pre-processing step because the collected images are clear before they reach the channel state. Furthermore, this creates a lively trade-off between frequency signals, which is visible at peak values. Due to the employment of Fourier filters, a transformation strategy using sequential resolution is used, with a large window size being recommended. Furthermore, utilizing a single matrix form, the time resolution of various signals will be exploited to separate positive and negative samples. Both positive and negative signals will be normalized with a combination factor after the pre-processing stage, as shown in Figure 3.

The separation of data from time scales that twitch from −2 to +3 when the transformation technique is adopted is depicted in Figure 3, Table 2. Additionally, the same amount of positive and negative samples are treated as identical situations when compared to reference inputs. After three state observations, the final combined rate for both the traditional and projected methods is calculated from signal images. Once the signals have been agreed upon, the arrival rate should be matched to the time scales and maximized.

**Table 2:** Combined rates of samples.

**Time scale**	**Positive samples**	**Negative samples**	**Combined rate (1)**	**Combined rate (Proposed)**
−2	21	10	147	219
−1	24	6	213	345
0	17	3	254	423
1	13	2	314	497
2	11	2	368	536
3	8	2	416	589

If the maximum rate is not obtained, it means that some stages of signal separation are not being processed, implying that loop depiction must begin at the beginning. According to the contrasted combined rate values, the projected technique employing deep learning models optimizes the rate at 589 signals per second, whereas the existing method can only maximize up to 416 signals per second, which is significantly less than the planned adaption.

### Scenario 3

**Figure 4:**
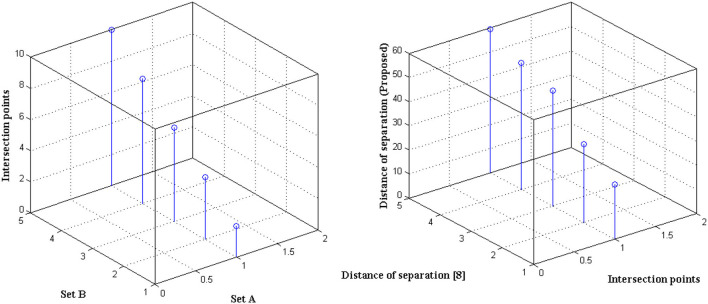
Separation of distance in the incidence of data set.

The distance of monitoring between numerous variations is an important case study after the representation of time scale in biological signals. The suggested technique considers four discrete samples, with the point of inter-section providing the actual distance. When the separation distance increases by a positive factor, it is referred to as scaling with regard to the time factor since the value of the signal representation changes. Furthermore, if deep learning is not used, the procedure might be defined as manual because positive variables must be multiplied by time scales. To avoid such manual procedures, a point of inter-section is chosen that has no effect on frequency variations. However, this method is limited since every 30 s inter-section interval, signal rotation is required. During this 30 s time period, over 1,000 signals are sampled within the channel without any data set augmentation. Figure 4 depicts the imprecise distance separation values that were simulated.

Figure 4, Table 3 show that two sets of values were chosen as input, the first of which differs by two value points and the second of which differ by four. These two sets are combined, and a point of inter-section is provided, as well as values at each midway, resulting in automatic distance configuration. For biological signals, a sensing device must be placed at each midway to maximize the separation distance. If the separation distance is not maximized, data will be augmented, which is a severe flaw in traditional models without deep learning techniques. However, the proposed system model solves it using five different points of touch, reducing the number of sensing devices from ten to five. Furthermore, 5 sensing devices are sufficient for viewing the representation signal at all phases, as evidenced by the last data set, where the suggested method achieves a distance of separation of 66.9, whilst the conventional methodology achieves a distance of separation of 59.5.

**Table 3:** Distance of separation.

**Set 1**	**Set 2**	**Distance of separation (8)**	**Distance of separation (Proposed)**
2	4	22.6	23.4
4	8	32.4	38.7
6	12	47.8	53.1
8	16	52.3	62.4
10	20	59.5	66.9

### Scenario 4

**Figure 5:**
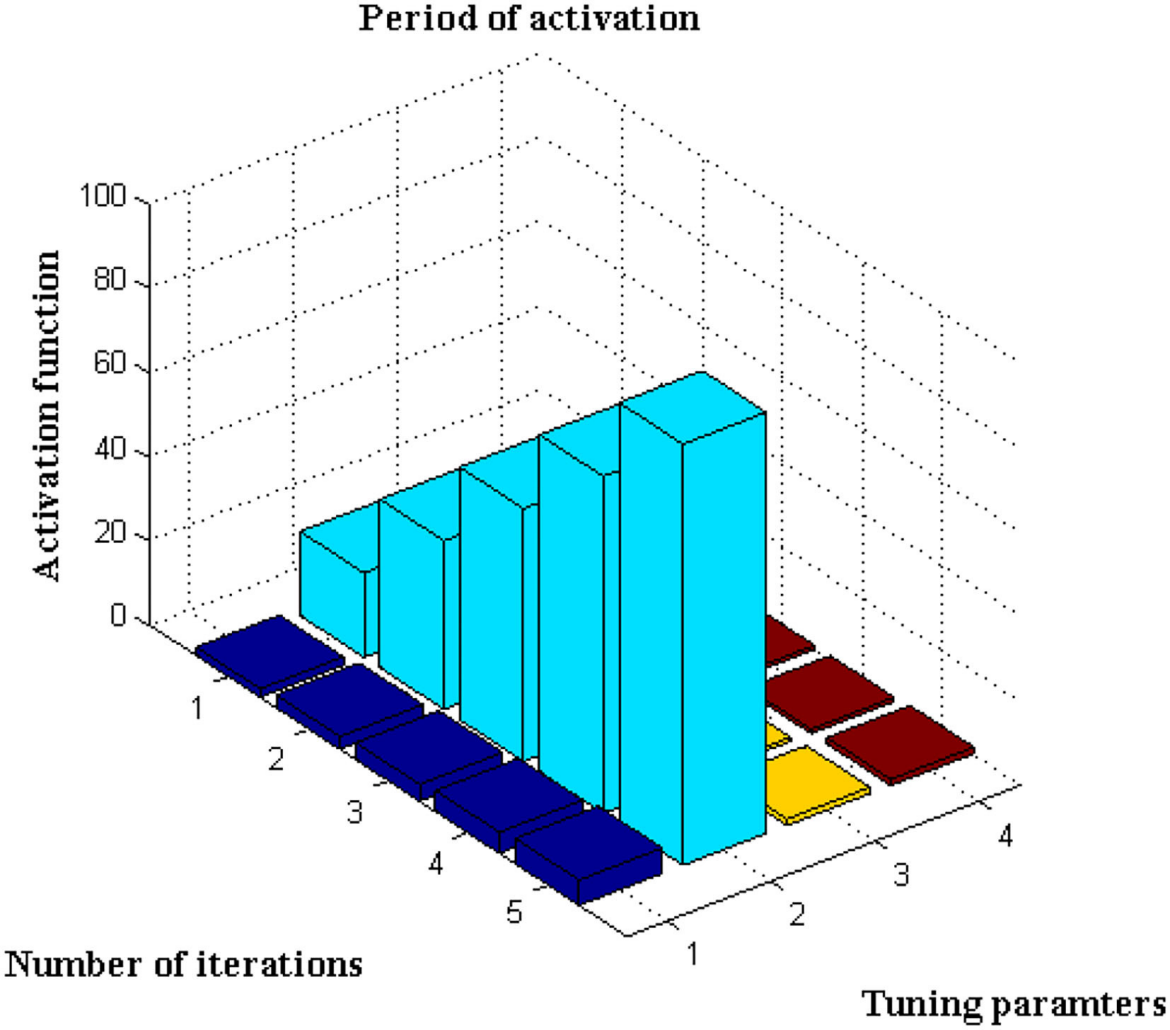
Activation function with iteration periods.

This scenario is implemented utilizing the activation function, which is represented using Equation, for further analysis of the data set (13). The number of iterations considered in this sort of initiation varies for each defined data set. It is characterized as an exact network if the activation period is obtained at negligible iteration, hence the maximum iteration period is set to 100 with a variation of 20 predetermined periods. The main rationale for choosing 20 defined periods is that during this sample, error values are decreased to 0.001, resulting in the best rate of concealed and output markers. Only if the following constraints are present, such as <4k Hz for variable activities and >30kHz for constant activities, will the training function be active. If any value exceeds a specific range, it means that the visual representation's border condition is incorrect, and testing data cannot be activated. After extensive testing, it was discovered that the proposed method's boundary conditions are met, and a visual representation is obtained, as shown in Figure 5.

Figure 5, Table 4 show that the bar that rises to a large extent is labeled as the activation period, whereas there is no rise in activation functions at three different stages. Aside from this activation function, the number of tuning parameters must be chosen in a ratio that is appropriate for the number of iterations. The proposed method increases the ratio to around 1:15, resulting in the best activation function within the time constraints. This is demonstrated at iteration 80, where a high activation function is achieved for 2.5 scaling periods. Because the proportion ratio in the existing model (9) is not properly defined, the activation period remains at 1 for even two iteration periods. This demonstrates that the projected deep learning model produces the best iteration results with the best activation functions.

**Table 4:** Iteration and activation function.

**Tuning parameters**	**Number of iterations**	**Activation function (9)**	**Activation function (Proposed)**
2	20	0.5	1.5
3	40	0.5	2
4	60	1	2
5	80	1	2.5
6	100	1.5	3

### Scenario 5

This Scenario depicts the cost of implementing observation units with hidden fragments. It is self-evident that the cost of implementation is decreased due to the minimal number of sensors deployed at junctions.

**Figure 6:**
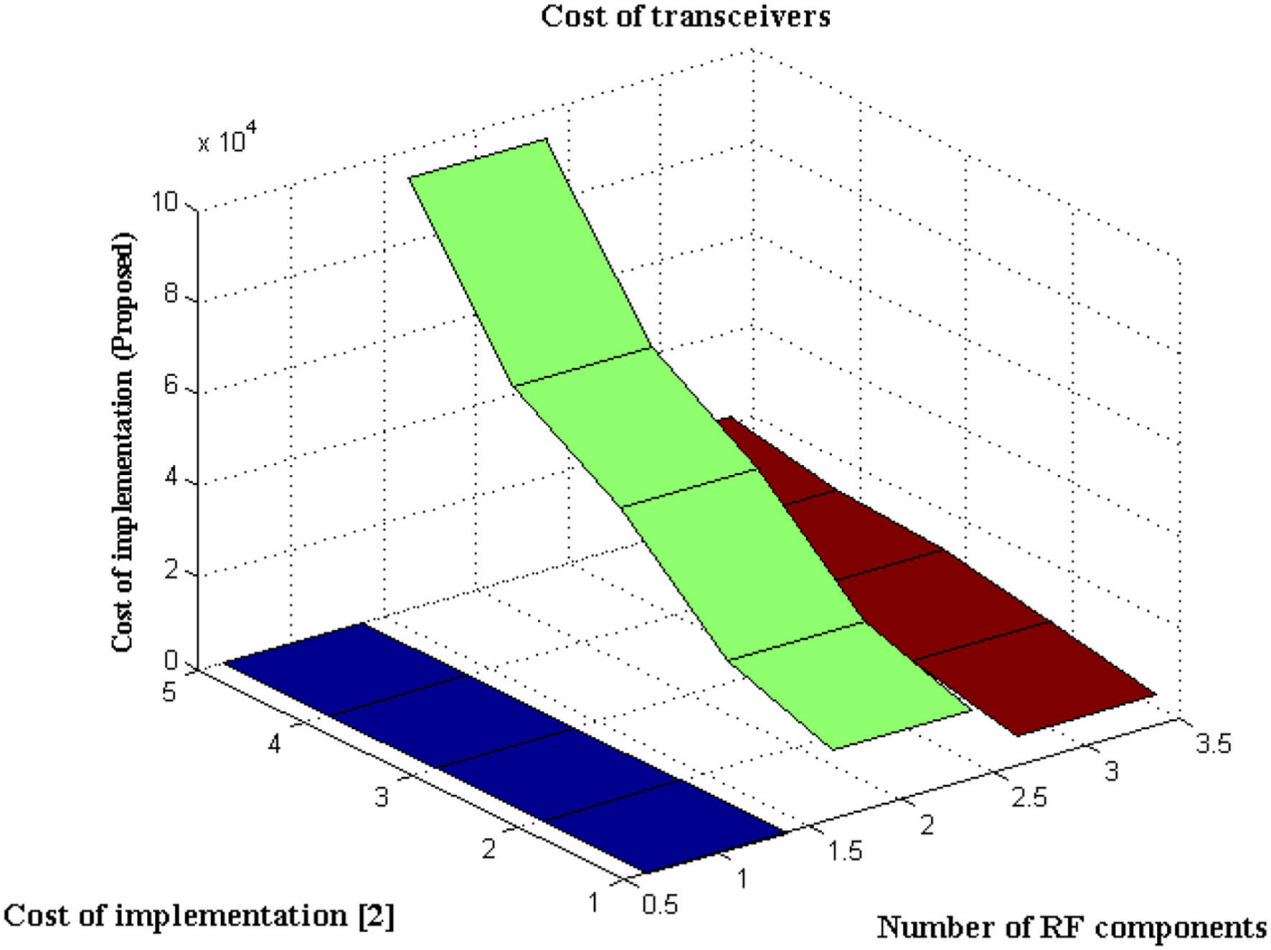
Implementation cost.

The type of transceiver used in the proposed technique is determined by the communication between different receivers and the control power of the stations. A radio frequency component is used as an external controller in the transceiver, and a series of recordings are made. This radio frequency component will raise the cost of signal acquisition, but a Nanoscale module is used in the deep learning process. Figure 6 depicts the simulated implementation cost with comparison. Figure 6 and Table 5 show that the cost of implementation increases as the number of radiofrequency components increases for both proposed and existing methods (2). However, with the same number of components, the existing method has a high implementation cost of INR 95000. However, the proposed method is implemented with INR21500 as the transceiver is designed properly at appropriate stages using the same components. Even the power constraint is met at the same cost of installation, resulting in cost savings. This demonstrates that the cost of real-time implementation may be significantly reduced, and medical enterprises can readily obtain it.

**Table 5:** Cost of existing and proposed methods.

**Number of RF components**	**Cost (2)**	**Cost (Proposed)**
3	15000	6700
6	23000	11300
9	45000	15600
12	60000	17200
15	94000	21500

### Performance Analysis

Since DCS are implemented in the proposed model it is essential to check the performance characteristics in addition to the above-mentioned scenarios. The major intention for checking the enactment is that DCS will have more than one output representation and in this case, the best output must be selected and it should be compared with reference data. This output representation wills ensemble the fitness function and it should be terminated after a certain number of algorithmic steps. Also, the implemented algorithm must provide high feasibility to all system representation techniques. Therefore three different performance analyses are carried out in this section as listed below,

#### Feasibility of DCS

In the proposed method a separate programming code is inscribed where step-by-step directions are included in the loop. The pre-defined loop features must be available with highly effective resources and a matching technique will be represented to check the allocation of resources at exact location points. Also, the probability distribution of a value must be defined with finite cases where both length and time margins are provided with an average value set.

**Figure 7:**
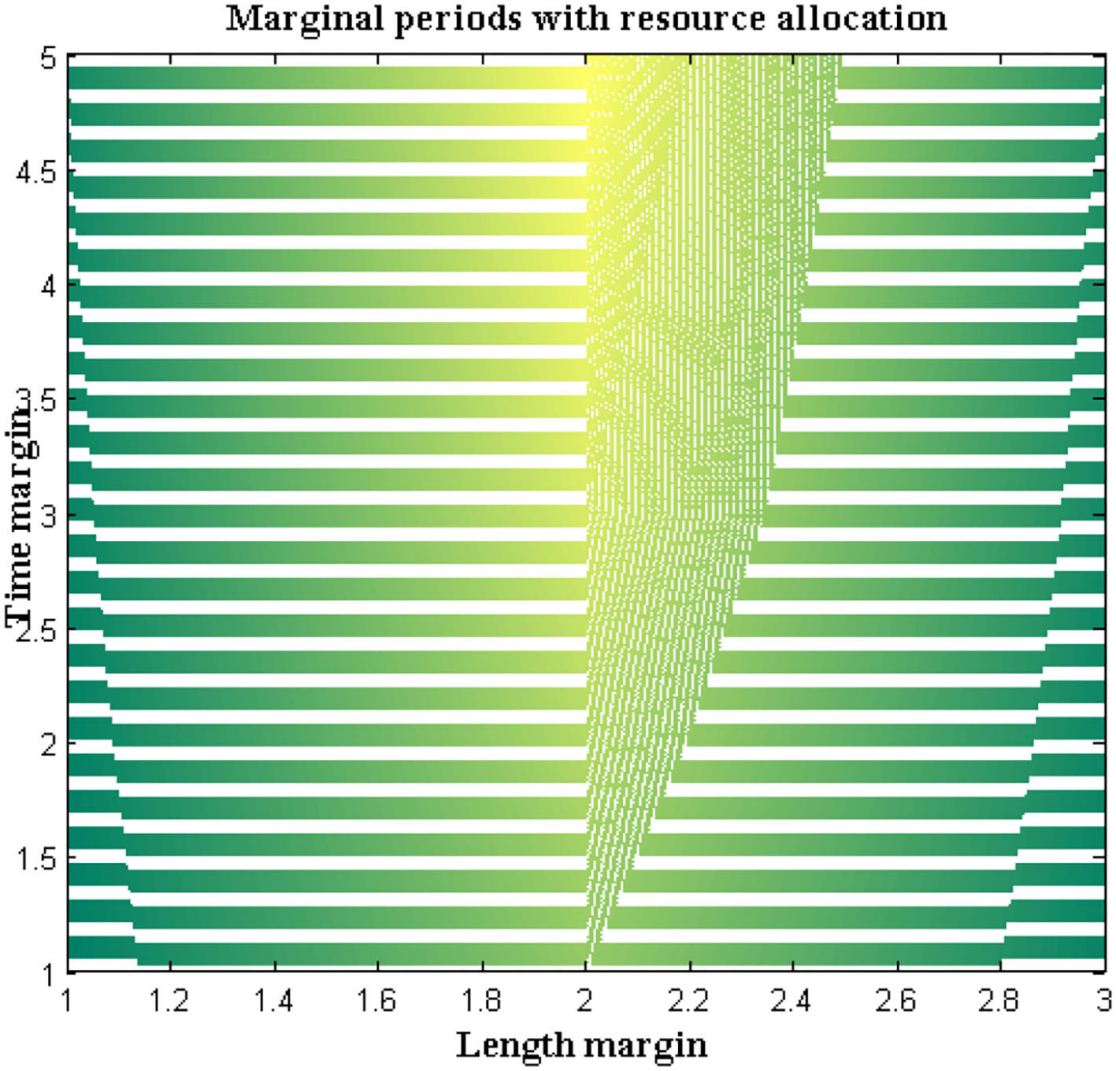
Marginal periods with the allocation of resources.

For all the data sets a non-trivial solution will be achieved with two different exponential functions. Additional sub-exponential functions are also derived to determine the performance of DCS using entropy function determinations where a feasible solution can be achieved. The accomplished solutions are deliberated in Figure 7. From Figure 7 it can be observed that a number of resources are varied from 4 to 20 and for each resource, both time and length margins are measured. For the proposed method both margins are kept as a minimum at the constraint for margins are satisfied as compared to reference values. However, comparisons are not made with any existing method due to unavailable resources in the selection process. Even the time margins which are represented in seconds are much lesser and the same can be applied to conjunction characteristics at border length margins. This can be proved with a maximum resource where length margins are 204 meters and, in this case, the maximum time period is 34 s. The above-mentioned values are much lesser as in the absence of resources more than 80 s time periods are observed.

#### Computational Complexity

**Figure 8:**
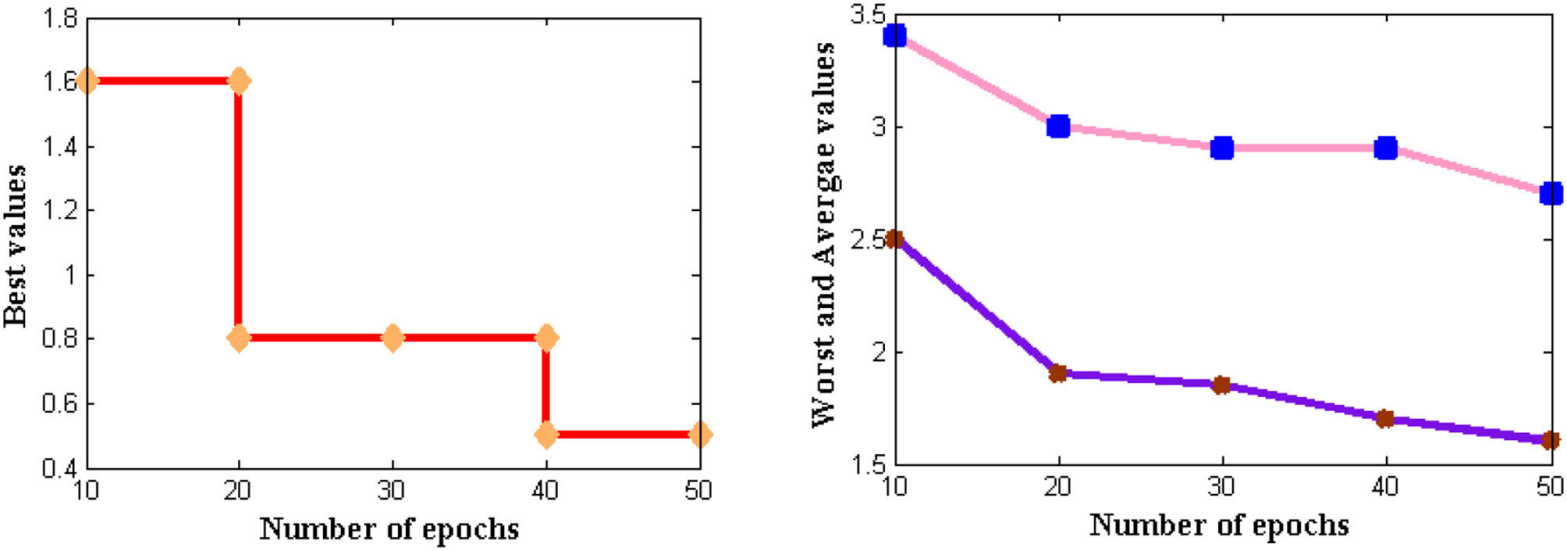
The complexity of time intervals.

If the feasibility of the proposed method is higher, then complexities that are present in time periods can be analyzed. Thus, complexities in time periods are measured in addition to memory requirements where for large input size three different values such as best, worst, and average are represented. In the projected model, the time for executing each statement is found and it is determined using the asymptotic function. The complexity in time periods is measured using several external factors such as the size of the input, processor speed, etc. The computational complexity for DCS is simulated in Figure 8.

From Figure 8 it can be observed that a number of iterations is considered from 10 to 50 and all three values are plotted where best values are shown in subplot 1, worst and average values in subplot 2. From these two subplots, DCS values are varied where low complexities are found as best values reach 0.5 s and further reduce after several iteration periods. If zero measurements are found then it indicates that time complexity is not present in the system. The same situation is observed for DCS after 80 iterations but until it reaches the corresponding iteration value complexities remain constant at 0.5 s. This proves that at constant speed DCS can able to reduce complexities in time.

#### Convergence Characteristics

**Figure 9:**
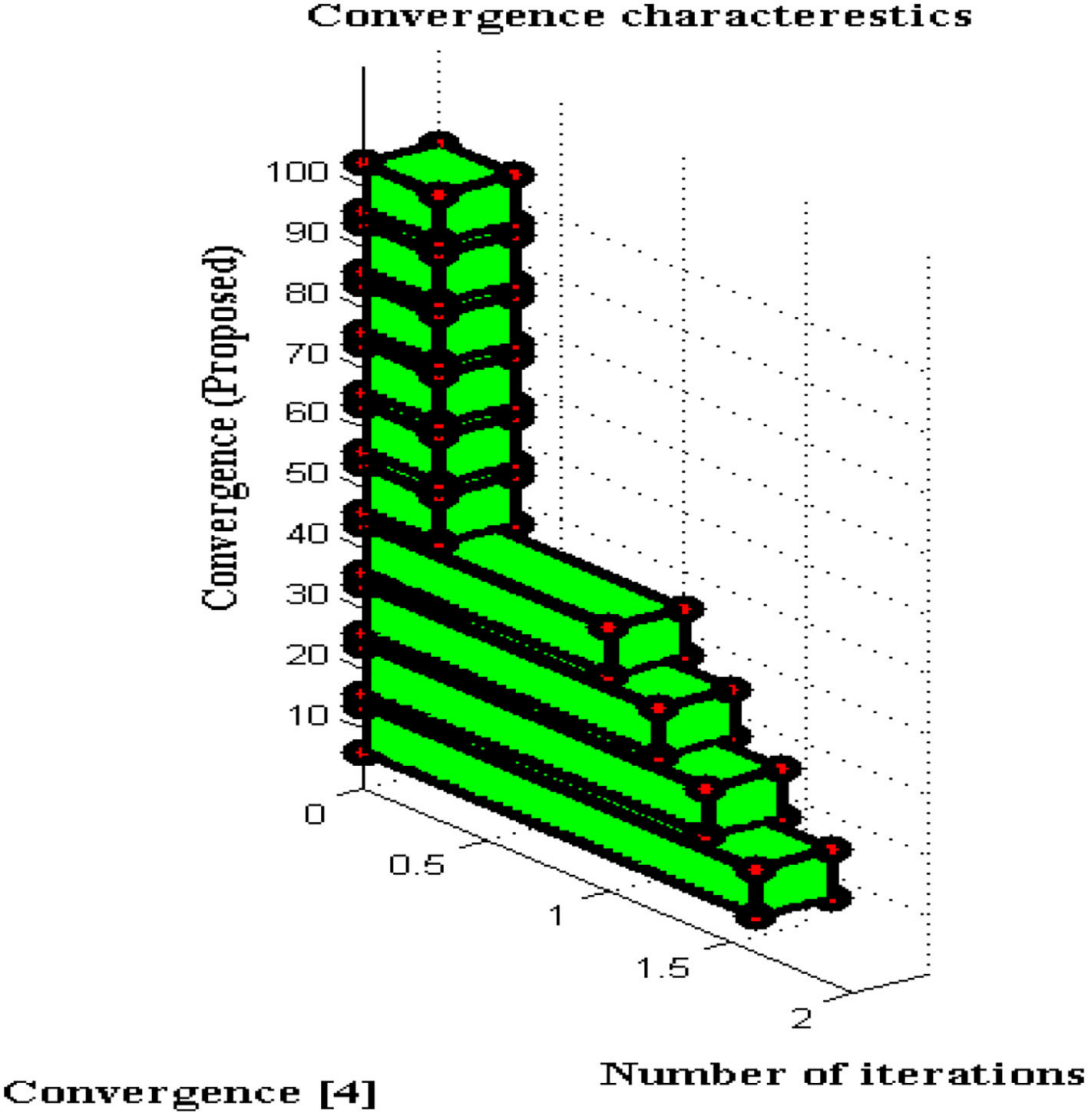
Convergence characteristics.

For all non-linear system models, it is essential to perceive the convergence characteristics where persistent phases must be observed. In DCS two types of characteristics are analyzed with respect to definite and captivating problems. This will give a clear solution to address the problems that arise during a long period of time where if convergence is not achieved then that particular time period will be separated. Furthermore, the separated time periods will be analyzed in several ways such as fault communication module, signal transmission properties, sensing capabilities, etc. If any one parameter fails to provide precise results then convergence cannot be achieved and it indicates failure of the model. The convergence characteristics of DCS are deliberated in Figure 9.

From Figure 9 it can be perceived that DCS is converged immediately at 50 iteration periods whereas the existing system converges only at 80 iteration periods. Therefore a large difference in convergence can be observed in the value determinations such as 0.3 and 0.5. Even after attaining fast convergence, the proposed method can be able to reduce the period of signal transfer to the receiver. This in turn helps the users to get a high signaling rate at shorter periods but fabrication parameters must have concurred with DCS. With the fabrication parameters, it is much easier to install a signal processing sensor thereby making DCS converged at initial point conditions.

#### Robustness of DCS

**Figure 10:**
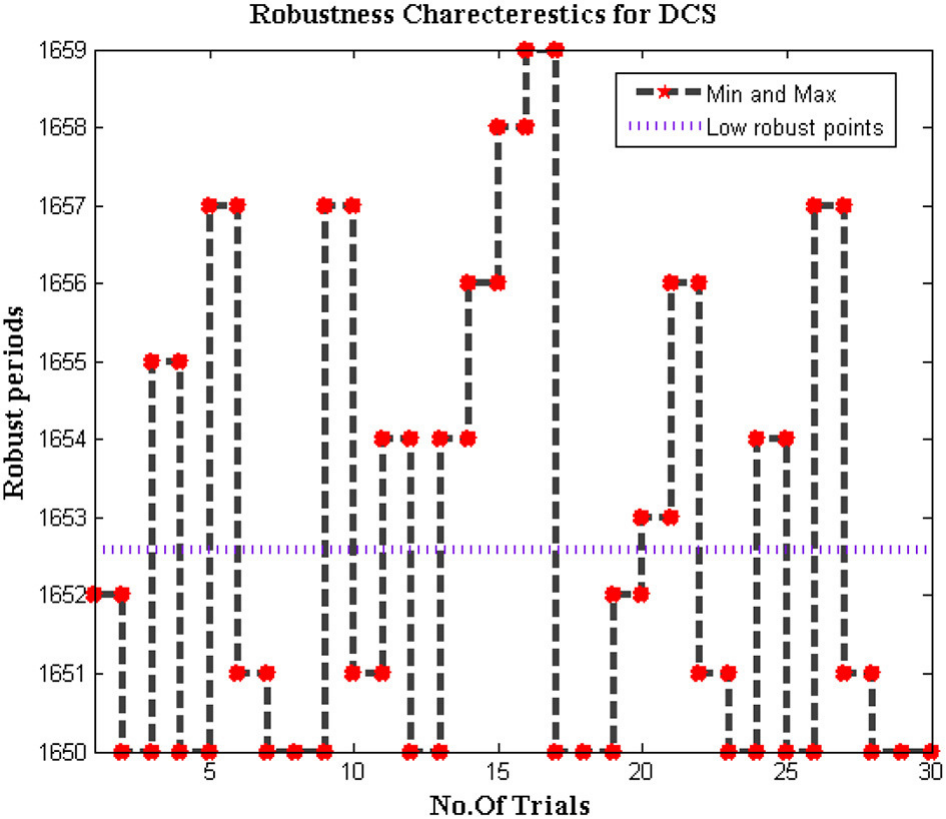
Low robust points of DCS.

Since the dataset of the proposed method is varied as different bio-medical signals are transferred to end systems. In addition, the biomedical signals that are passed from individuals are much different when it is compared with distinct parameters. Therefore for change in signal representation, it is essential to check whether the system is robust to all input conditions. Further error in DCS must be reduced to increase the effectiveness of the proposed model as the trained inputs must achieve much closer outcomes during deduction segments. Thus the simulated model for checking the robustness is plotted in Figure 10.

From Figure 10 it can be observed that the number of iterations varied from 10 to 100 and for each representation, a midpoint robust values are achieved. These midpoint values are considered as the initial design of the biomedical signal representation must not be changed as local points will provide information about missing values in the system. For all changes in iteration periods, the proposed method operates under low robust conditions whereas the conventional systems are highly robust against input conditions as local points cannot able to identify distinct data set points.

## Conclusions

This paper examines a real-time augmentation biomedical signal that is used in the testing and classification mechanism of a human interface system. The application of biomedical signals in medical applications provides clear insights into future developments under the automatic mode of operation in a wide range of research fields. As a result, the proposed method prepares an automatic process using a sensing device that can be accessed in simple environmental conditions. However, a few sensors that monitor biomedical signal parameters must be fabricated in order to provide precise data and transmit signal data to the control center. In the proposed method, a pre-processing technique is used at stage 1 to classify and train the images, with the result that any incongruent signals will be detected quickly. In addition, for the training set, the previous data set has been integrated, and a comparative analysis has been performed using two algorithms: firefly and gate count technology. Since biomedical signals in the transceiver are detected using a deep learning algorithm, which includes DCS with several learning parameters in the 2.5-time scale period range. As a result, the generated data set is observed for three discrete periods using weight transformations, resulting in deep image representation extensions.

Furthermore, real-time simulation results show that biomedical signals are activated without any peripheral intrusion, even after several different classifications. To gain a better understanding of DCS, five different scenarios are divided and tested using a hardware setup that yields effective results. Biomedical signals can be used as a building block for all knowledge transformation techniques in the future, resulting in a significant reduction in data augmentation measures.

## Data Availability Statement

The original contributions presented in the study are included in the article/supplementary files, further inquiries can be directed to the corresponding authors.

## Author Contributions

Data curation: AY. Writing-original draft: HM. Supervision: SS. Project administration, conceptualization, and visualization: SS and HM. Methodology and resources: SS and MU. Validation: HM, HA, and AY. Review, editing, and funding acquisition: C-LC and C-MW. All authors contributed to the article and approved the submitted version.

## Funding

This work was supported in part by the National Natural Science Foundation of China (No. 51808474) and the Ministry of Science and Technology in Taiwan (Nos. MOST 110-2218-E-305-001–MBK and MOST 110-2410-H-324-004-MY2).

## Conflict of Interest

The authors declare that the research was conducted in the absence of any commercial or financial relationships that could be construed as a potential conflict of interest.

## Publisher's Note

All claims expressed in this article are solely those of the authors and do not necessarily represent those of their affiliated organizations, or those of the publisher, the editors and the reviewers. Any product that may be evaluated in this article, or claim that may be made by its manufacturer, is not guaranteed or endorsed by the publisher.
